# Adherence to Pharmacological Treatment in Chronic Venous Disease: Results of a Real-World, Prospective, Observational Cohort Study

**DOI:** 10.3390/life15030377

**Published:** 2025-02-27

**Authors:** Daciana Elena Branisteanu, Alice Elena Munteanu, Bogdan Mihai Dolofan, Elena Gabriela Popescu, Oana Vittos

**Affiliations:** 1 Faculty of Medicine, General, Dermatology Discipline, University of Medicine and Pharmacy “Gr. T. Popa”, 700115 Iasi, Romania; 2 Central Military Emergency University Hospital “Dr. Carol Davila”, 010825 Bucharest, Romania; 3 Medical Affairs Department, Servier Pharma, 013714 Bucharest, Romania; 4 Clinical Research Department, Medone Research, 18, Alexandr Sergheevici Puskin Street, District 1, 011996 Bucharest, Romania

**Keywords:** treatment adherence, chronic venous disease, epidemiology, symptomatology, CEAP, MMAS-8

## Abstract

Chronic venous disease (CVeD) affects millions of patients globally, being a multifactorial progressive condition that significantly impacts the quality of life of individuals. Micronized Purified Flavonoid Fraction (MPFF) is the most utilized and studied venoactive drug because of its safety and effectiveness. This study is a real-world, prospective, observational, multicenter cohort study including patients diagnosed with CVeD who were receiving one tablet of MPFF 1000 mg/day for at least one month and who visited medical facilities in Romania in June–July 2022. We aimed to assess their adherence to pharmacological treatment. The intensity of CVeD symptoms was assessed with the Visual Analog Scale (VAS), while adherence to conservative treatment was evaluated using the Morisky Medication Adherence Scale (MMAS-8) at study inclusion (Visit 1 (V1)) and 8 weeks later, at the study’s end (Visit 3 (V3)). This study recruited 1267 patients diagnosed with CVeD, and the statistical analysis set included 1200 patients, the majority of whom were female (71.5%), ≥51 years old (81.8%), and overweight (41.2%) or obese (33.8%), with a mean Body Mass Index (BMI) value (±SD) of 28.9 ± 5.1 kg/m^2^, classified using the Clinical, Etiological, Anatomical, and Pathophysiological (CEAP) clinical categories as CEAP C3 (38.7%) and C2 (22.6%) at baseline. Mean MMAS-8 scores increased from 6.2 ± 1.9 (V1) to 6.7 ± 1.7 (V3) (*p* < 0.001). Despite improvement in treatment adherence throughout this study, novel strategies are needed to improve medication adherence and overall health outcomes in CVeD.

## 1. Introduction

Chronic venous disease (CVeD) affects millions of patients globally and is a progressive condition that significantly impacts the quality of life of individuals and their families. It represents a significant healthcare cost, which is projected to rise in the next decades as a consequence of population aging and the constant increase in obesity prevalence [1,2,3,4]. CVeD prevalence is estimated to vary across different demographic and occupational groups. A scoping review reported high prevalence rates among healthcare workers due to prolonged standing and physical strain [5], while another study highlighted the utility of validated electronic health records in understanding the epidemiology of CVeD in primary care settings [6]. These findings underscore the widespread impact of CVeD and the importance of tailored management strategies.

Conservative treatment for CVeD mainly consists of compression therapy and Venoactive Drugs (VADs) [4], among which the most utilized and studied class for safety and effectiveness is Micronized Purified Flavonoid Fraction (MPFF), which is a combination of 90% diosmin and 10% other flavonoids (diosmetin, hesperidin, linarin, and isorhoifolin), expressed as hesperidin.

MPFF treatment increases venous tone and has antioxidant and venous anti-inflammatory properties, inhibiting leukocyte–endothelium interaction [7]. At the same time, MPFF increases capillary resistance and lymphatic drainage and decreases capillary permeability [8]. In clinical trials, the administration of 1000 mg/day of MPFF for 2 months in patients with CVeD classified according to the Clinical, Etiological, Anatomical, and Pathophysiological (CEAP) classification as clinical category CEAP C0 (subjects presenting leg symptoms without visible signs of CVeD) was linked to the elimination of the transitory commissural reflux [9].

Adherence to medication is an essential part of patient care, and currently, there are no data available for Romania regarding adherence levels to conservative treatment in adult outpatients diagnosed with CVeD who are treated with MPFF 1000 mg/day.

## 2. Materials and Methods

This real-world, longitudinal, multicenter, prospective, observational cohort study was conducted in Romania in adult outpatients diagnosed with CVeD who were under conservative treatment for at least one month with one tablet of MPFF 1000 mg/day and who had visited a general practitioner, dermatologist, or surgeon between 20 June and 17 July 2022. All of the patients included signed the informed consent form approved by the Ethics Committee.

A total of 81 investigators were included from the healthcare system of Romania. Each investigator was allowed to enroll a maximum of 20 patients in this study. The investigators established and maintained the CVeD treatment as per their best medical judgment, compliant with current medical practice guidelines and in the best interests of the patient.

The main objective of this study was to evaluate the CVeD patients’ adherence to conservative treatment and medical recommendations by using the Morisky Medication Adherence Scale—8 (MMAS-8). The secondary objectives were to collect and evaluate information about lifestyle and risk factors, to evaluate the venous symptoms of the lower limbs (“heaviness”, “leg pain”, “sensation of swelling”, “cramps”) by measuring the intensity of these symptoms evaluated by patients using a Visual Analog Scale (VAS) (at visits V1 and V3), and to evaluate the management of routine medical care for patients with CVeD who were already being treated with MPFF 1000 mg/day.

This study included adult outpatients diagnosed with chronic venous disease (CVeD) receiving one tablet of MPFF 1000 mg/day for at least one month. This study excluded children from participation, as well as patients who had visited the medical unit under medical emergencies and patients with other conditions that might have prevented participation in this study, such as limited cooperation, limited legal capacity, other serious illnesses or conditions that affected life expectancy (cancer, drug abuse, etc.) or serious cardiovascular disease, liver failure, and kidney failure.

This study consisted of three visits: an inclusion visit (V1), a telephone follow-up visit (V2) after 4 weeks, and a second follow-up visit (V3) at 8 weeks. During this study, the following data were collected: demography (age, gender, weight, height), current CVeD symptomatology and symptom intensity evaluated using the VAS, the CEAP clinical stages of CVeD [10], lifestyle (physical activity level, smoking status, prolonged sitting or standing position), the presence of risk factors for CVeD (accidental/surgical injuries, thrombophlebitis, blood clotting disorders, etc.), and treatment adherence measures using the MMAS-8 questionnaire. Objective methods for assessing swelling (e.g., leg circumference measurements or ultrasound) were not included due to the observational nature of this study and the constraints of real-world clinical practice. BMI was calculated automatically in eCRF based on the patient’s weight and height data collected by the investigator.

MMAS-8 scores were also analyzed according to the CVeD CEAP class and age group. In addition to the MMAS-8 questionnaire, questions regarding compliance with the venoactive treatment recommendations were asked by investigators at each visit. This study collected data regarding patients’ and physicians’ satisfaction with CVeD pharmacological treatment.

The MMAS-8 is used for assessing patient adherence to a certain treatment through a list of 8 questions to which the patient responds with “yes” or “no” (questions 1–7), while the last item is a 5-point Likert response. Patients’ answers are dependent on their personal experience, without the influence of the investigator or other parties. MMAS-8 scores can range from zero to eight, and a scoring algorithm can be obtained from the developer [11].

Statistical analysis was performed with SPSS version 21 (SPSS Inc., Chicago, IL, USA) and included descriptive analysis and tests employed to determine the importance of distribution differences in the researched variables, involving a paired samples T-test for quantitative variables and a Chi-square test for categorical variables.

As this observational study aimed to capture real-world data, no formal sample size calculation was conducted prior to its initiation. Instead, the sample size was determined based on the anticipated availability of eligible patients during the recruitment period, which was considered adequate to investigate the objectives of this study. This study focused on evaluating adherence and compliance in a real-world setting, where patient behaviors and decisions naturally influenced outcomes.

The scale variables were reported as the mean value and standard deviation (SD), and categorical variables were reported using frequencies and percentages. All values that were missing or considered incorrect were handled as missing without any mathematical model for data imputation. A *p*-value < 0.05 was considered indicative of a statistically significant difference.

This observational study received approval from the National Bioethics Committee for Medicine and Medical Devices (NBCMMD), number 6SNI, dated 24 February 2022, and the National Agency of Medicine and Medical Devices in Romania (NAMMDR) was notified regarding this study, with the notification number 2014E, dated 7 June 2022.

## 3. Results

**Figure 1:**
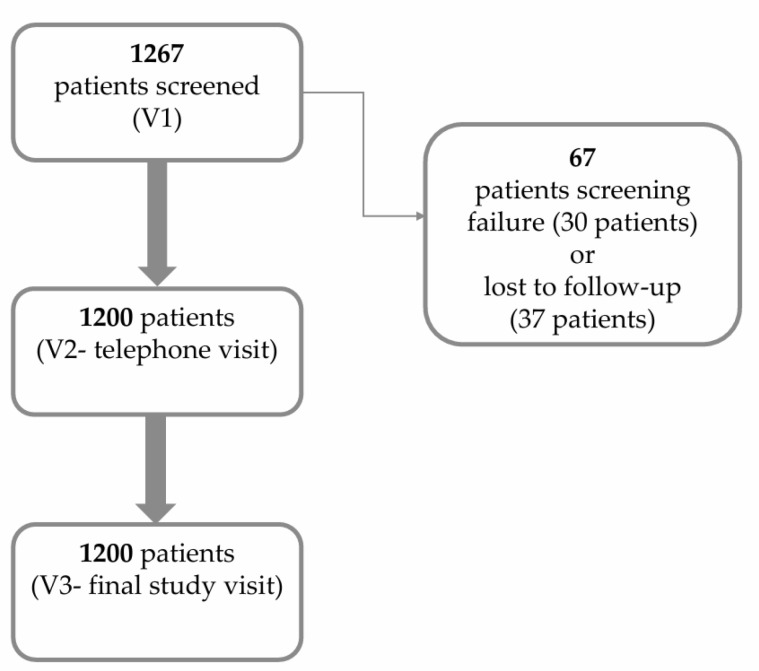
Study flow chart C.

This study enrolled 1267 patients. Only 1200 of these patients complied with the study inclusion criteria and attended all study visits; they were included in the analysis set (30 patients excluded due to screening failure and 37 patients lost to follow-up) (Figure 1). The majority were female (71.5%), 51 years old or older (81.8%), and overweight (41.2%) or obese (33.8%), with a mean Body Mass Index (BMI) value (±SD) of 28.9 ± 5.1 kg/m^2^.

The following risk factors were present for most of the patients: sedentary lifestyle or with moderate physical activity (81.8%), prolonged orthostatic position (≥5 h/day) (41.8%), prolonged standing position (≥5 h/day) (56.3%), CVeD family history (57.0%), and concomitant presence of other venous diseases (35.3%) (Table 1).

The most frequent concomitant diseases were arterial hypertension (70.4%) and dyslipidemia (48.0%). More than half of the study patients (57.8%) had one or more risk factors for thrombosis most frequently reported, such as thrombophlebitis (27.1%) or accidental/surgical injuries (19.3%) (Table 1).

At baseline, most patients were categorized within the CEAP C3 class (38.7%), followed by the CEAP C2 class (22.6%) and CEAP C4a (15.8%) (Table 2).

At inclusion, the most frequent CVeD symptoms were “heaviness”, reported by 1096 patients (91.3%), followed by “sensation of swelling”, reported by 1023 patients (85.3%), and “leg pain”, reported by 1029 patients (85.8%). Most frequently, the symptoms occurred at the end of the day (79.0%) or after long periods of orthostatic position (57.2%). A similar distribution of CVeD symptomatology, in a decreased proportion, was observed at the end of this study (Table 3).

**Figure 2:**
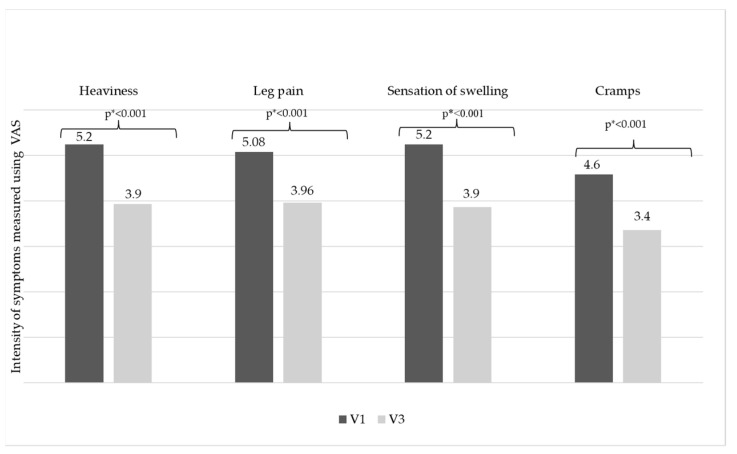
CVeD symptomatology evolution throughout this studyCaption. CVeD = Chronic Venous Disease; * Wilcoxon Signed Rank Test.

After 8 weeks, the intensity for all CVeD symptoms measured with the VAS decreased significantly (from V1 to V3): “Heaviness” 5.2 ± 2.2 cm vs. 3.9 ± 2.1 cm; “leg pain” decreased 5.1 ± 2.3 cm vs. 3.9 ± 2.1 cm; “sensation of swelling” 5.2 ± 2.4 cm vs. 4.0 ± 2.2 cm; and the intensity of “cramps” 4.6 ± 2.3 cm vs. 3.4 ± 2.1 cm (*p* < 0.001) (Figure 2).

At inclusion, 467 (38.9%) patients were considered to show low adherence (MMAS-8 score < 6), 371 (30.9%) medium adherence (MMAS-8 score between 6 and 8), and only 362 (30.2%) high adherence (MMAS-8 score = 8). At V3, adherence increased significantly; low, medium, and high adherence was observed in 342 (28.5%), 351 (29.3%), and 507 patients (42.3%), respectively (*p* < 0.001) (Table 4).

**Figure 3:**
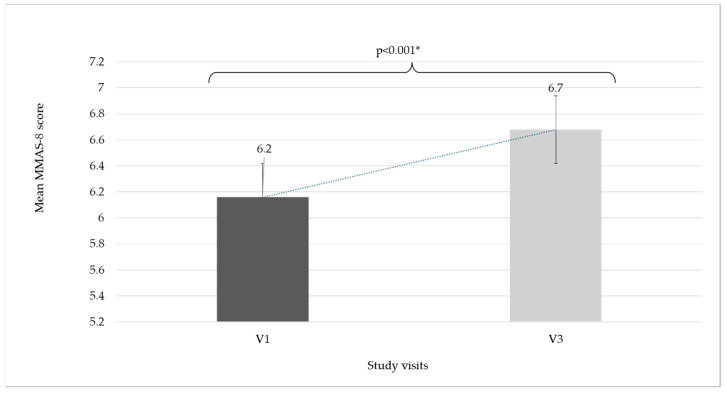
Mean MMAS-8 score evolution during this study. * Wilcoxon Signed Rank Test, MMAS-8 = Morisky Medication Adherence Scale—8.

At baseline, the mean MMAS-8 score was 6.2 ± 1.9 and increased significantly throughout this study to 6.7 ± 1.7 (V3) (*p* < 0.001); nevertheless, despite this increase, it should be noted that the mean MMAS-8 score remained suboptimal, with a medium value throughout this study (Figure 3).

**Figure 4:**
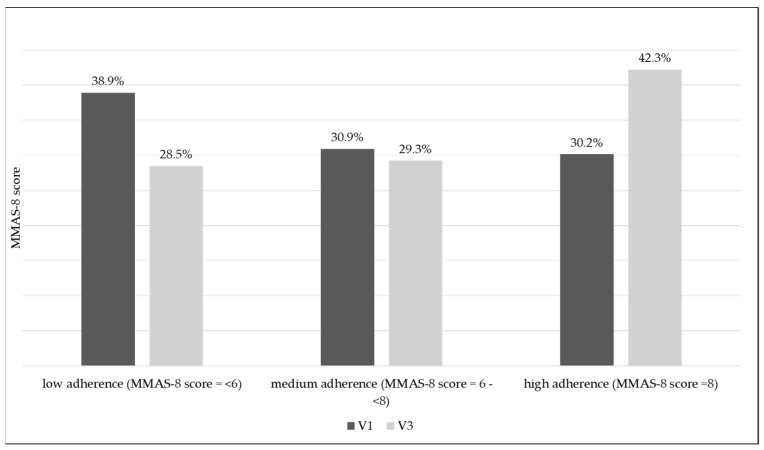
MMAS-8 score evolution during this study. MAS-8 = Morisky Medication Adherence Scale—8.

Only 42.3% of patients reached optimal adherence at V3 (Figure 4).

When the MMAS-8 scores were assessed for each CEAP class, it was observed that an increase in MMAS-8 score was maintained throughout this study for all of the CEAP classes (Table 4).

**Figure 5:**
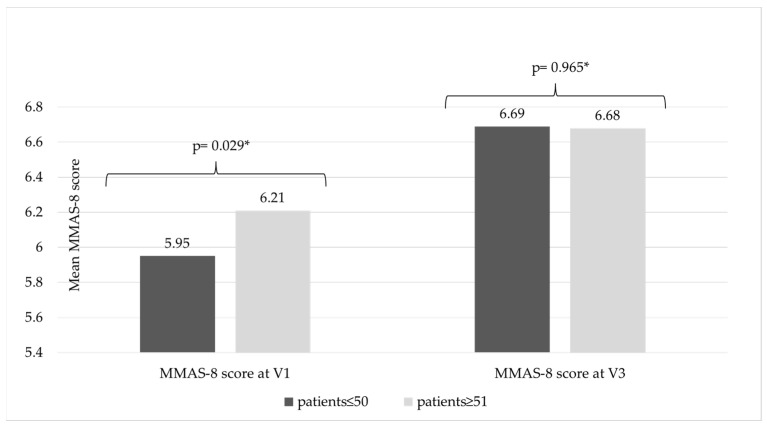
Pharmacological treatment adherence based on age group. * Wilcoxon Signed Rank Test.

CVeD pharmacological treatment adherence was assessed in younger individuals versus older patients, with a cut-off age of 50 years. It was noted that, at study inclusion, the mean MMAS-8 scores were significantly lower in the younger patients’ group (5.9 ± 1.9) versus the older group (6.2 ± 1.9) (*p* = 0.029); however, this difference was not maintained during this study (Figure 5).

Questions regarding compliance with the venoactive treatment recommendations were asked by investigators at each visit, in addition to the MMAS-8 questionnaire. When the question was asked by the investigator, the patients showed a clear tendency to answer in a positive manner, with 96.3% of patients declaring they were compliant at V3; however, when the patients completed the MMAS-8 questionnaire, the answers were quite different, and at V3, only 68.8% of patients declared that they did not forget to take the medication (Table 5).

For patients skipping medication, the most frequently reported reason was “unintentionally” or “forgetfulness” (Table 6).

Patients’ and physicians’ satisfaction with CVeD pharmacological treatment consisting of one tablet per day of MPFF 1000 mg was analyzed, and 47.8% of patients and 49.0% of physicians were very satisfied with the conservative treatment. More than 50.0% of patients declared they were either very motivated or extremely motivated to continue the treatment (Table 7).

Besides pharmacological treatment with MPFF 1000 mg/day, 99.0% of patients received lifestyle advice, while 66.3% (796 patients) received recommendations for compression therapy. The use of compression therapy increased according to the CEAP class, from 16.67% for the C0 class to 100% for the C6 class (Table 8).

## 4. Discussion

Adherence to medication is an essential part of patient care, and compliance with treatment is necessary to reach therapeutic goals. The World Health Organization (WHO) 2003 report underlined the capital importance of treatment adherence, mentioning that “increasing the effectiveness of adherence interventions may have a far greater impact on the health of the population than any improvement in specific medical treatment” [12].

This is the first real-world study, performed in Romania, that aimed to evaluate the adherence to conservative treatment in adult outpatients diagnosed with CVeD treated with MPFF 1000 mg/day using the MMAS-8, together with the intensity of CVeD symptomatology evaluated with the VAS.

It has been demonstrated that treatment with MPFF improves signs and symptoms of CVeD. The systematic reviews of double-blind placebo-controlled trials demonstrated that MPFF is highly effective in improving symptoms in patients with CVeD. MPFF is characterized by powerful anti-inflammatory and venoprotective effects and might have the potential to alter the clinical course of CVeD by preventing and delaying CVeD complications [5,13,14,15,16,17,18,19].

As per international treatment guidelines, MPFF is recommended as the reference treatment for the management of CVeD [20,21]. A dose/effect ratio of MPFF has been identified with a linear relationship between the logarithm of the MPFF dose and the effect on venous hemodynamics, and it seems that the best dose/effect ratio is achieved with 1000 mg/day of MPFF, which means at least 900 mg/day of diosmin [22].

The patient population included in this study was considered representative of the Romanian CVeD population, consisting mainly of females (71.5%), with the majority being aged 51 years and older (81.8%), overweight (41.2%), or obese (33.8%). The demographic data, corroborated with the risk factors, were similar to those in other epidemiological studies (Vein Consult Program or Vein Act Program) [23,24]. The age categories (<30, 30–50, and >50 years) were selected to represent distinct demographic groups that may exhibit different adherence behaviors and clinical characteristics. This categorization allowed for the exploration of potential age-related differences in treatment adherence and symptomatology, which can provide valuable insights into tailored management strategies for patients with chronic venous disease.

The threshold of 5 h of sitting or standing was selected in alignment with previous studies [25,26] and based on its association with venous stasis and chronic venous disease progression. A comparison between two standing tests (20 min of quiet standing vs. 5 h of quiet sitting) showed increases in blood pressure and in total peripheral resistance, decreases in heart rate (7 beats/min), cardiac output (12.7%), and calf blood flow (18.5%), and a 70% increase in calf venous pooling after sitting [27]. Additionally, data from worldwide surveillance of self-reported sitting time showed that the median of mean daily sitting times was 4.7 (IQR: 3.5–5.1) hours across all countries. Higher-income countries recorded a longer duration of sitting time than lower-income countries (4.9 vs. 2.7 h) [28].

In this study, as per the specific inclusion criterion, all patients included received conservative pharmacological treatment, namely one tablet of MPFF 1000 mg/day, for at least one month prior to participating in this study. This, at the end of the observational period, is equivalent to a minimum length of 3 months of conservative treatment, directed by each treating physician, according to his/her medical judgment, as per practice guidelines and in the best interest of the patients. The treatment included MPFF 1000 mg/day for all patients, compression therapy (66.3% of patients), and lifestyle advice (99% of patients).

According to the current edition of Management of Chronic Venous Disorders of the Lower Limbs: Guidelines According to Scientific Evidence, “the MPFF retained its strong recommendation for use as adjuvant therapy in treating venous leg ulcers” [4,29], and based on the meta-analysis results of five randomized clinical trials involving 723 patients with venous ulcers, it states that “at six months, the chance of ulcer healing was 32% better in patients treated with the combined therapy than those managed by compression alone. It was noticed that this difference was present from month two and was associated with shorter time to healing, with level of evidence high (Grade A)” [4,30].

In our study, the use of compression therapy varied between CEAP classes, from 16.67% (C0) to 100% (C6), and the overall figures of compression therapy use are higher compared to other reports from international studies [25].

This study demonstrated that, despite a medium treatment adherence detected using MMAS-8 scoring throughout the observational period, MPFF 1000 mg/day is an important treatment option, alone or combined with other conservative treatment options, for CVeD. It relieves symptoms, showing clinically and statistically decreasing CVeD symptom intensity measured with the VAS during the 8-week observational period, where the VAS scores decreased from 5.2 ± 2.2 cm (V1) to 3.9 ± 2.1 cm (V3) for “heaviness” symptoms; from 5.1 ± 2.3 cm (V1) to 4.0 ± 2.1 cm (V3) for “leg pain”; from 5.2 ± 2.4 cm (V1) to 3.9 ± 2.2 cm (V3) for “sensation of swelling”; and from 4.6±2.3 cm (V1) to 3.7 ± 2.1 cm (V3) for “cramps” (*p* < 0.001). Given that patients were under MPFF treatment for at least one month, the improvement noticed within our study might be because MPFF treatment had not reached optimal effectiveness by the time of study inclusion, possibly due to the low patient adherence to treatment prior to study inclusion, adherence that, despite improving during this study, remained suboptimal.

Randomized studies demonstrated that one tablet of MPFF 1000 mg/day has a similar safety and efficacy profile to two tablets of MPFF 500 mg/day [31,32,33]. The advantage of once-per-day administration of a certain medication might be linked to higher improvement in patient adherence to CVeD pharmacotherapy.

In this study, adherence to MPFF 1000 mg/day pharmacotherapy was evaluated with the MMAS-8 questionnaire. The MMAS-8 is a validated instrument that could be used to identify patients with treatment-related adherence problems, which should be corrected and monitored during treatment. This aspect is especially important for chronic disease treatment.

The MMAS-8 instrument is a self-reported scale that assesses medication adherence [34,35]. Since its development in 2008, the MMAS-8 has been used in more than 200 studies [34].

The MMAS-8 has been shown to have acceptable internal consistency and reproducibility [34]. As an indirect measurement of patient adherence to drug treatment, it has been shown to have external validity for the prediction of outcomes, including poorer blood pressure control or higher HbA1c measurements in patients on antihypertensive [36] or antidiabetic therapy [37], respectively. The predictive value of the MMAS-8 has not been confirmed for selected outcomes in all studies [11,34,38].

Patient self-reported adherence scales aim to measure medication-taking behavior and can help identify barriers to adherence in patients [35]. The MMAS-8 is one of two current scales with the highest level of evidence [39]. It has been studied in a range of patient settings in addition to hypertension and diabetes, including cancer, inflammatory bowel disease, and Parkinson’s disease [34,40]. To the best of our knowledge, this is the first use of the MMAS-8 in chronic venous disease.

Despite the limitations associated with any psychometric instrument, the extensively studied MMAS-8 provides important, novel information about medication adherence in the study population.

It is known that approximately 50–60% of patients do not adhere to prescribed medication [41,42]. Similarly, the current study shows that despite CVeD being a symptomatic disease, at inclusion, only 61.1% of patients were considered to show medium or high adherence to CVeD conservative treatment. It was observed that adherence increased significantly throughout this study; at the end of the study, only 28.5% continued to show low adherence, while 71.5% of patients showed medium or high adherence (*p* < 0.001). It is worth mentioning that, at baseline, the lowest MMAS-8 score was identified for both CEAP C0 and C6 patients, those who did not adhere to CVeD treatment. At the end of this study, all CEAP classes had mean MMAS-8 scores between 6 and 8, values indicative of medium adherence. This might be explained by physician intervention at study inclusion and during telephone visits, where the importance of patient compliance with medical treatment was reinforced, together with high reported patient and physician satisfaction with the one tablet per day MPFF 1000 mg treatment.

Data collected at inclusion revealed that treatment adherence scores in the younger population were significantly lower compared to the older population; however, this trend was not maintained during the study. These data are similar to those of other published research, where younger patients reported higher nonadherence rates compared to older patients [43]. Additional studies will be needed to identify factors influencing treatment adherence in younger patients with chronic pathologies.

In the present study, the declared patient compliance with the CVeD conservative treatment of one tablet a day of MPFF 1000 mg was higher than the numbers found within the literature concerning CVeD conservative treatment with two tablets of MPFF 500 mg per day [22,23], whereas the other mentioned studies did not use the MMAS-8 for the evaluation of treatment adherence.

For our study, a significant increase in MMAS-8 values from 6.2 ± 1.9 (at inclusion) to 6.7 ± 1.7 (end of study) was observed (*p* < 0.001). Nevertheless, despite the medical staff intervention and a relatively short follow-up period (8 weeks), it should be mentioned that the MMAS-8 scores remained suboptimal, and at least one out of four patients continued to show low adherence to conservative CVeD pharmacotherapy.

For this observational study, certain limitations should be considered, namely, being a descriptive study and focusing on real-world evidence collection rather than hypothesis testing. The study sample size was determined by the availability of eligible participants in a real-world, multicentric setting, ensuring representativeness, and not based on formal calculation. Additionally, there were some variables that were not collected consistently throughout this study from all patients, and some were missing. Due to the real-world, observational nature of this study, the updated CEAP classification (2020) [44] was not used, as it was not broadly adopted by all physicians involved when the study was conducted. Additionally, there are potential sources of bias regarding the interpretation of results due to the potential inaccuracy of CEAP classification performed by physicians or the potential inaccuracy of self-reported behaviors of patients.

## 5. Conclusions

This real-world evidence study performed in Romania demonstrated that during this short-term observation period, less than half of the patients were highly adherent to conservative CVeD pharmacotherapy (42.3%), with one out of four patients continuing to show low adherence, proving that there is an urgent need for the early identification of non-compliant patients, who require additional support. Novel strategies to improve medication adherence and overall health outcomes in CVeD are needed.

## Figures and Tables

**Table 1:** Patients’ baseline characteristics.

**Gender, *N* (%)**	***N* = 1200**
Female,	858 (71.5%)
Male	342 (28.5%)
**Age, *N* (%)**	***N* = 1200**
<30 years	8 (0.7%)
30–50 years	210 (17.5%)
51–70 years	607 (50.5%)
>70 years	375 (31.3%)
**Weight (kg), Mean ± SD**	80.4 ± 15.4
**Height (cm), Mean ± SD**	162.3 ± 6.5
**BMI (kg/m^2^), Mean ± SD**	28.9 ± 5.1
**BMI, *n* (%)**	***N* = 1200**
<18.5 (underweight)	8 (0.7%)
18.5–25 (normal weight)	263 (21.9%)
25–30 (overweight)	485 (40.4%)
30–40 (first grade obesity)	413 (34.4%)
>40 (second grade obesity)	31 (2.6%)
**Physical activity, *n* (%)**	***N* = 1200**
Sedentary	439 (36.6%)
Moderate physical activity	542 (45.2%)
Intermediate physical activity	206 (17.1%)
Intense physical activity	13 (1.1%)
**Prolonged sitting position, *N* (%)**	***N* = 742**
<5 h/day	424 (57.1%)
5–10 h/day	318 (42.9%)
**Prolonged standing position, *N* (%)**	***N* = 758**
<5 h/day	371 (48.9%)
5–10 h/day	387 (51.1%)
**Smoker, *n* (%) (*N* = 1200)**	192 (16.0%)
**CVeD family history, *N* (%)**	***N* = 1200**
One parent	523 (43.6%)
Both parents	111 (9.2%)
Grade 2 relatives	50 (4.2%)
No medical history of CVeD	516 (43.0%)
**Risk factors for thrombosis, *N* (%)**	***N* = 1200**
Accidental/surgical injuries	231 (19.3%)
Prolonged immobilization in bed (postpartum or surgical)	49 (4.1%)
Long journeys at altitude or in a sitting position	107 (8.9%)
Thrombophlebitis	266 (22.2%)
Blood clotting disorders	40 (3.3%)
None of the above	591 (49.2%)
**Concomitant diseases, *N* (%)**	***N* = 1200**
Hypertension	845 (70.4%)
Ischemic Coronary Disease (ICD)	337 (28.1%)
Type 2 Diabetes Mellitus (T2DM)	192 (16.0%)
Obesity	444 (37.0%)
Dyslipidemia	576 (48.0%)
Others	366 (30.5%)
**Female population**	***N* = 858**
Number of births (%- reported to female study population), *n* (%)	
0	85 (9.9%)
1	301 (35.1%)
2	348 (40.6%)
>2	124 (14.5%)
Use of hormone replacement therapy (%- reported to female study population), *n* (%)	10 (1.2%)
Use of birth control pills (%- reported to female study population), *n* (%)	16 (1.9%)

*N* = Analyzed set; *n* = number of non-missing observations; BMI = Body Mass Index; VeD = Chronic Venous Disease.

**Table 2:** CEAP classification of CVeD at study inclusion visit.

CEAP Class (*N* = 1200)	V1	
	*n*	%
C0s	6	0.5%
C1	77	6.4%
C2	271	22.6%
C3	464	38.7%
C4a	190	15.8%
C4b	88	7.3%
C5	64	5.3%
C6	40	3.4%

*N* = Analyzed set; *n* = number of non-missing observations; CEAP = Clinical (C), Etiological (E), Anatomical (A), and Pathophysiological (P); CVeD = Chronic Venous Disease.

**Table 3:** Evolution of CVeD symptoms.

CVeD Symptoms, *n* (%)	V1	V3
Heaviness	1096 (91.3%)	1002 (83.5%)
Leg pain	1029 (85.8%)	937 (78.1%)
Sensation of swelling	1023 (85.3%)	906 (75.5%)
Cramps	875 (72.9%)	715 (59.6%)
**Time when CVeD symptoms appear at highest intensity, *N* = 1200**	**V1**	
	***n***	**%**
At the end of the day	948	79.0%
After long periods of orthostatic position	686	57.2%
During the night	353	29.4%
After a long period of sitting	234	19.5%

*N* = Analyzed set; *n* = number of non-missing observations; CEAP = Clinical (C), Etiological (E), Anatomical (A), and Pathophysiological (P); CVeD = Chronic Venous Disease.

**Table 4:** MMAS-8 score evolution.

Treatment Adherence According to MMAS-8	V1		V3		*p*-Value (V1–V3) *
	*n* (%)	Mean ± SD	*n* (%)	Mean ± SD	
**low (MMAS-8 score < 6)**	467 (38.9%)	4.1 ± 1.3	342 (28.5%)	4.4 ± 1.4	*p* < 0.001
**medium (MMAS-8 score 6–8)**	371 (30.9%)	7.0 ± 0.5	351 (29.3%)	7.0 ± 0.5	*p* < 0.001
**high (MMAS-8 score > 8)**	362 (30.2%)	8.0 + 0.0	507 (42.3%)	8.0 + 0.0	*p* < 0.001
**Mean MMAS score**	6.2 ± 1.9		6.7 ± 1.7		*p* < 0.001
**CEAP class**	**V1**		**V3**		*p*-value (V1–V3) *
	***n* (%)**	**MMAS-8 score (Mean ± SD)**	***n* (%)**	**MMAS-8 score (Mean ± SD)**	
**C0**	6 (0.5%)	5.8 ± 1.9	8 (0.7%)	6.3 ± 2.2	0.269
**C1**	77 (6.4%)	6.2 ± 2.0	89 (7.4%)	6.6 ± 2.0	0.001
**C2**	271 (22.6%)	6.4 ± 1.8	326 (27.2%)	6.9 ± 1.6	<0.001
**C3**	464 (38.7%)	6.3 ± 1.8	425 (35.4%)	6.7 ± 1.7	<0.001
**C4a**	190 (15.8%)	6.2 ± 1.8	192 (16.0%)	6.6 ± 1.6	<0.001
**C4b**	88 (7.3%)	5.3 ± 2.0	61 (5.1%)	6.1 ± 2.0	<0.001
**C5**	64 (5.3%)	6.1 ± 1.9	67 (5.6%)	6.5 ± 1.6	0.001
**C6**	40 (3.4%)	5.3 ± 2.3	32 (2.7%)	6.7 ± 1.7	<0.001

* Wilcoxon Signed Rank Test; CEAP = Clinical (C), Etiological (E), Anatomical (A), and Pathophysiological (P).

**Table 5:** Treatment adherence.

Direct and Indirect Questions and Answers	V1		V3	
	Yes	No	Yes	No
Patients’ answers to the question asked verbally during the visit by the investigator regarding compliance with venoactive treatment recommendations, *n* (%) *	1142 (95.2%)	38 (3.2%)	1156 (96.3%)	21 (1.8%)
Patients answers to the question asked in the MMAS-8 questionnaire: Do you sometimes forget to take your medication(s)? *n* (%)	465 (38.8%)	735 (61.3%)	375 (31.3%)	825 (68.8%)

* Certain answers are missing.

**Table 6:** Declared reasons for skipping medication.

Reasons for Skipping the Treatment (V3) Declared by Patient	*n*	%
Forgot to take the medicine on certain days	98	74.2%
Ran out of medicine	11	8.3%
Takes too much medicine	9	6.8%
Does not take every day, only when needed	3	2.3%
Another reason	11	8.3%

**Table 7:** Patients’ and physicians’ feedback at the end of this study regarding CVeD conservative treatment.

Patient and Physician Satisfaction with Conservative Treatment Result	Patient Satisfaction	Physician Satisfaction
0—Dissatisfied	2 (0.2%)	3 (0.3%)
1—Rather dissatisfied	20 (1.7%)	18 (1.5%)
2—Rather satisfied	325 (27.1%)	271 (22.6%)
3—Very satisfied	574 (47.8%)	588 (49.0%)
4—Extremely satisfied	279 (23.3%)	320 (26.7%)
**Patient motivation to follow recommended treatment**	***N***	**%**
0—Not motivated	2	0.2%
2—Rather unmotivated	27	2.3%
4—Rather motivated	155	12.9%
6—Motivated	313	26.1%
8—Very motivated	427	35.6%
10—Extremely motivated	276	23.0%

**Table 8:** Compression therapy.

CEAP Classification			Patient Receiving Compression Therapy at V1 (*n* = 796)		
CEAP Class	*n*	% (Reported to Total Study Population, *N* = 1200)	*N*	% (Reported to Total Study Population, *N* = 1200)	% (Reported to CEAP Class)
C0	6	0.50%	1	0.08%	16.67%
C1	77	6.42%	21	1.75%	27.27%
C2	271	22.58%	152	12.67%	56.09%
C3	464	38.67%	314	26.17%	67.67%
C4a	190	15.83%	150	12.50%	78.95%
C4b	88	7.33%	67	5.58%	76.14%
C5	64	5.33%	51	4.25%	79.69%
C6	40	3.33%	40	3.33%	100.00%
Total	1200	100%	796	66.33%	

## Data Availability

The datasets generated and/or analyzed during the current study are not publicly available because they belong to Servier, as the sponsor of this study, but are available from the corresponding author upon reasonable request, and with prior permission of Servier.

## Abbreviations

The following abbreviations are used in the manuscript:

BMI	Body Mass Index
CEAP	Clinical (C), Etiological (E), Anatomical (A), and Pathophysiological (P)
CVeD	Chronic Venous Disease
ICD	Ischemic Coronary Disease
MMAS-8	Morisky Medication Adherence Scale—8
MPFF	Micronized Purified Flavonoid Fraction
NAMMDR	National Agency of Medicine and Medical Devices in Romania
NBCMMD	National Bioethics Committee for Medicine and Medical Devices
T2DM	Type 2 Diabetes Mellitus
VADs	Venoactive drugs
VAS	Visual Analog Scale
